# Gepirone for Major Depressive Disorder: From Pharmacokinetics to Clinical Evidence: A Narrative Review

**DOI:** 10.3390/ijms26199805

**Published:** 2025-10-08

**Authors:** Natalia Gałka, Emilia Tomaka, Julia Tomaszewska, Patrycja Pańczyszyn-Trzewik, Magdalena Sowa-Kućma

**Affiliations:** 1 Department of Human Physiology, Faculty of Medicine, University of Rzeszów, Al. Tadeusza Rejtana 16C, 35-959 Rzeszów, Poland; 2 Student’s Science Club of Physiology “NEURON”, Faculty of Medicine, University of Rzeszów, Al. Tadeusza Rejtana 16C, 35-959 Rzeszów, Poland; 3 Centre for Innovative Research in Medical and Natural Sciences, Collegium Medicum, University of Rzeszów, Warzywna 1a, 35-310 Rzeszów, Poland

**Keywords:** gepirone, EXXUA, major depressive disorder, 5-HT1A agonist, antidepressant, anxiety, pharmacology

## Abstract

Gepirone, a selective 5-hydroxytryptamine (serotonin) 1A (5-HT_1A_) receptor agonist, offers a promising strategy for treating mood and anxiety disorders. The therapeutic importance of 5-HT_1A_ modulation is well established, as these receptors regulate serotonergic neurotransmission both presynaptically, in the somatodendritic regions of raphe neurons, and postsynaptically, in structures including the hippocampus, neocortex, septum, amygdala, and hypothalamus. Gepirone exhibits a distinctive pharmacological profile, acting as a full agonist at presynaptic autoreceptors and a partial agonist at postsynaptic receptors, with high affinity for 5-HT_1A_ and much lower affinity for 5-HT_2A_ receptors. Its effects on serotonergic signaling are time-dependent. Acute administration suppresses serotonergic firing through autoreceptor activation, while chronic treatment induces autoreceptor desensitization, leading to enhanced 5-HT release in projection areas. This process is complemented by partial agonism at postsynaptic 5-HT_1A_ receptors, which further supports long-term neuromodulation. This article provides an integrated overview of gepirone’s mechanism of action, bridging receptor pharmacology, neurophysiological adaptations, and therapeutic implications. Particular emphasis is placed on the compound’s unique dual role in regulating serotonergic tone over time, a feature that differentiates it from other 5-HT_1A_-targeting agents. By linking molecular mechanisms to clinical outcomes, we highlight gepirone’s potential advantages in efficacy, safety, and tolerability compared with conventional antidepressants. This comprehensive perspective underscores gepirone as a paradigmatic example of selective 5-HT_1A_ modulation and offers novel insights into the development of targeted treatments for depression and anxiety.

## 1. Introduction

In 2022, the World Health Organization (WHO) sounded the alarm, estimating that 280 million people worldwide were living with depressive disorders, commonly referred to as depression [1]. Among these, major depressive disorder (MDD) stands out as the most prevalent form [2]. Moreover, epidemiological data indicate that by 2030, MDD will become the leading cause of disability in society, both in terms of health and social impact [1,2]. Notably, suicidality is strongly associated with the pathogenesis of MDD. A comprehensive review by Cui et al. revealed that individuals with MDD are at a significantly higher risk of suicidality compared with non-MDD groups [2,3].

From a clinical perspective, the most common symptoms of MDD include persistent sadness, low mood, and loss of interest in activities previously enjoyed [4]. Patients also experience low energy, impaired concentration, difficulties maintaining a healthy sleep cycle, and disturbed appetite. Overall, these symptoms may lead to a wide range of difficulties, such as an inability to manage everyday responsibilities [5,6]. Despite the well-defined clinical symptomatology of this disorder, the diagnosis of MDD is often difficult to establish at the first examination. This difficulty is related, among other factors, to the co-occurrence of somatic symptoms (known as masked depression) and the lack of specific biomarkers that could significantly facilitate the diagnostic process [7]. Another challenge for modern psychiatry is the limited effectiveness of commonly used antidepressants [8,9]. Pharmacological treatment of MDD usually involves serotonin and norepinephrine reuptake inhibitors (SNRIs) or selective serotonin reuptake inhibitors (SSRIs), but these approaches have several drawbacks, such as delayed onset of therapeutic effects, lack of response in some patients, and poor overall efficacy. Moreover, side effects can make it difficult for patients to continue treatment [9,10]. For this reason, increasing research efforts are focused on identifying novel compounds with antidepressant properties, characterized by new or more specific mechanisms of action [11,12,13,14].

**Figure 1:**
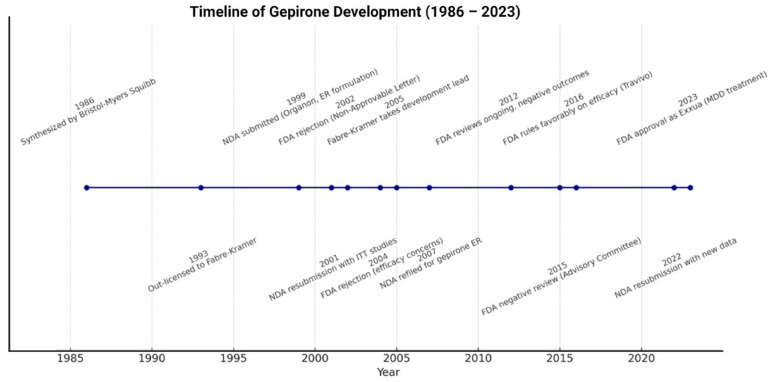
Timeline of gepirone development (Created in Biorender, M.S.-K. (2025) https://www.biorender.com/w23tz2w).

A growing body of evidence identifies gepirone as an innovative option in the pharmacological management of MDD. The compound has been under investigation since the early 1980s (originally labeled MJ-13805; also known as BMY-13805, ORG-33062 or Travivo) [15,16], but its development pathway has been unusually long and complex. Initially synthesized by Mead Johnson and later developed by Bristol-Myers for anxiety and depression, gepirone was first tested in an immediate-release (IR) form. However, its clinical potential was limited by extensive first-pass metabolism in the liver and low bioavailability. To overcome these drawbacks, research shifted toward a once-daily extended-release (ER) formulation with more favorable pharmacokinetics [17]. In 1993, Fabre-Kramer Pharmaceuticals Inc. obtained rights to gepirone ER. Five years later, Organon, Inc. took over development and commercialization, but in 2005 full ownership returned to Fabre-Kramer. The compound’s regulatory journey was similarly challenging. The FDA rejected approval applications in both 2002 and 2004. A new drug application (NDA) was resubmitted in 2007, later supplemented in 2009, yet in 2012 the FDA again ruled against approval, citing insufficient efficacy evidence for anxiety and depression. A further negative opinion followed in 2015 regarding its use in depression. Interestingly, in March 2016 the FDA reversed course and provided a favorable stance, which ultimately led to final approval in September 2023 (for review see Figure 1). Under the trade name EXXUA^®^ (ER tablets for oral use), gepirone was authorized in the United States for the treatment of MDD, supported by two pivotal efficacy trials (FK-GBE-007 and 134001) [18,19,20,21].

What differentiates gepirone from other antidepressants is its unique serotonergic mechanism of action, centered on the modulation of pre- and postsynaptic 5-HT1A receptors (will be described in detail later in this review). While structurally related to buspirone, gepirone does not interact with dopamine (D) receptors [22]. This property reduces the likelihood of adverse effects commonly associated with dopaminergic activity, such as extrapyramidal symptoms (dystonia, akathisia), prolactin elevation, or treatment-emergent sexual dysfunction often seen with certain antipsychotics and antidepressants [22,23,24,25]. Importantly, compared to buspirone, gepirone exhibits a longer half-life, the possibility of once-daily dosing in its ER formulation, and a stronger evidence base in the treatment of MDD rather than anxiety disorders alone [15,26]. Relative to tandospirone (other azapirone), which is mainly approved in Japan and China for anxiety and depression but has a short elimination half-life and limited global use, gepirone offers a more favorable pharmacokinetic profile and broader clinical validation [27,28]. In turn, unlike to perospirone (combines partial 5-HT1A agonism with potent antagonism at D2 and 5-HT2A receptors), gepirone is highly selective for 5-HT1A receptors [29,30]. This selectivity minimizes the risk of antipsychotic-like side effects while ensuring a targeted antidepressant effect. Collectively, these properties position gepirone as the most selective and clinically advanced azapirone for the treatment of MDD.

Given the limited efficacy and tolerability of standard antidepressants, the search for novel therapeutic options remains an urgent priority in psychiatry. In this context, gepirone, with its unique serotonergic profile, warrants a comprehensive evaluation to clarify its clinical potential and therapeutic positioning. The objective of this review is to critically assess the role of gepirone in the treatment of MDD and anxiety disorders, drawing on the full spectrum of available evidence, including pharmacodynamic and pharmacokinetic properties, drug interactions, preclinical findings (in vitro and in vivo), and clinical investigations (phases I–III and observational studies). We further compare the immediate-release (IR) and extended-release (ER) formulations, assess efficacy and safety profiles, highlight potential clinical advantages and limitations, and identify gaps and priorities for future research. A structured literature search was conducted in PubMed/MEDLINE, Embase, Web of Science, and Scopus for articles published between 1983 (the first report on gepirone) and the present. The following terms and their combinations were used: “gepirone”, “MJ-13805”, “BMY-13805”, “ORG-13011”, “Exxua”, “depression”, “major depressive disorder”, “MDD”, “anxiety”, “generalized anxiety disorder”, “GAD”, “panic disorder”, “pharmacokinetics”, “pharmacodynamics”, “extended-release”, “immediate-release”, “ER”, “IR”, “metabolism”, “CYP3A4”, “efficacy”, “safety”, “randomized”, “clinical trial”, “preclinical”, “animal”. Case reports and non0-peer-reviewed sources were excluded from the analysis. Only articles published in English were considered. The final reference list was compiled based on originality, methodological quality, and relevance to the broad scope of this review, with priority given to peer-reviewed preclinical and clinical studies providing data on the pharmacology, efficacy, and safety of gepirone.

## 2. Pharmacology

**Figure 2:**
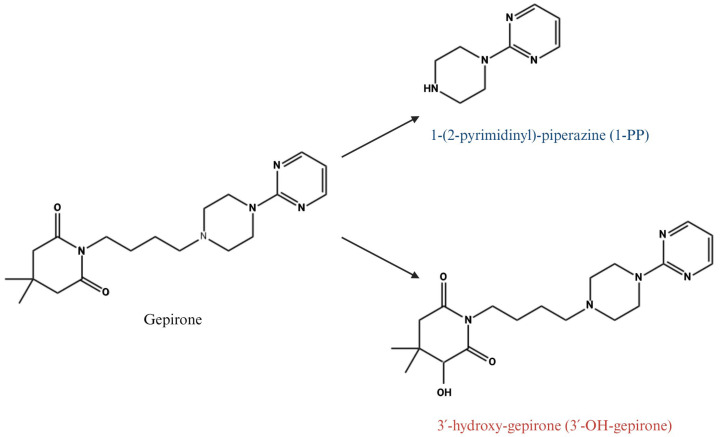
Molecular structure of gepirone and its two principal metabolites (Created in Biorender. M.S.-K. (2025) https://biorender.com/om58vha).

Gepirone, an azapirone derivative, is chemically classified as *2,6-piperidinedione*, *4,4-dimethyl-1-[4-[4-(2-pyrimidinyl)-1-piperazinyl]butyl]-*, *monohydrochloride*. This unique molecular structure, with a molecular weight of 359.5 g/mol and a molecular formula of C_19_H_29_N_5_O_2_ (Figure 2) [31], distinguishes it from other drugs in its class. The earliest clinical investigations employed the IR formulation, which was later replaced by the ER version. The ER formulation offers improved pharmacokinetic properties and has demonstrated greater clinical utility, ultimately leading to its approval for the treatment of MDD under the trade name EXXUA^®^ [31].

The pharmacological mechanism of gepirone has not yet been fully elucidated. Current evidence suggests that its antidepressant effects are primarily mediated through modulation of serotonergic neurotransmission, particularly via interactions with 5-HT_1_A receptors [32]. Notably, this mode of action differs from that of standard antidepressant classes such as SSRIs and SNRIs, underscoring its distinct receptor-binding profile (described in detail below) [33].

### 2.1. Pharmacodynamics

**Figure 3:**
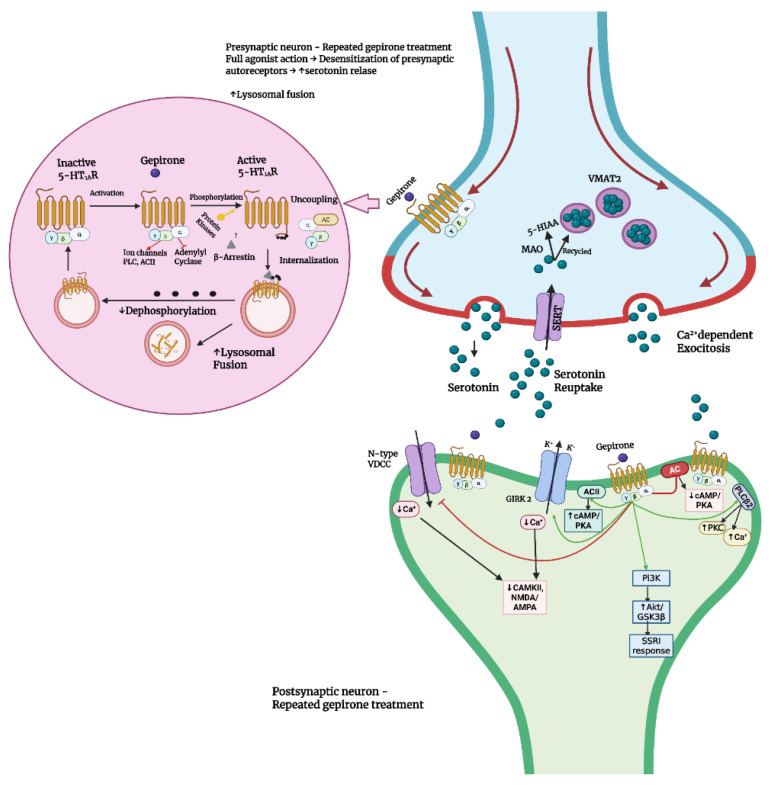
Schematic representation of gepirone’s mechanism of action at the serotonergic synapse. (Created in Biorender, N.G. (2025) https://BioRender.com/joffy1x). Gepirone exhibits high selectivity for 5-HT_1_A receptors, acting as a full agonist at presynaptic autoreceptors in serotonergic neurons and as a partial agonist at postsynaptic receptors located in brain regions such as the hippocampus, cortex, and amygdala. Repeated administration of gepirone leads to desensitization of presynaptic autoreceptors (this process involves receptor phosphorylation, β-arrestin recruitment, internalization, and lysosomal fusion), resulting in increased 5-HT release into the synaptic cleft via calcium-dependent exocytosis. In the postsynaptic neuron, gepirone activates 5-HT1A receptors, modulating cAMP/PKA, PI3K, and MAPK/ERK signaling pathways. These changes regulate ion channel activity and gene expression, contributing to its long-term antidepressant and anxiolytic effects.

The antidepressant and anxiolytic properties of gepirone involve modulation of serotonergic neurotransmission, mediated by a unique combination of receptor-binding affinities [34]. The receptor-binding profile of gepirone has been primarily characterized in preclinical studies, which identified it as a selective 5-HT_1A_ receptor agonist [35]. The therapeutic relevance of this mechanism is well established, as 5-HT_1A_ receptor agonists are involved in neuromodulation and can effectively alleviate symptoms of depression and anxiety [36]. These receptors are distributed in two principal locations: presynaptically, in the somatodendritic regions of raphe neurons, and postsynaptically, in brain structures such as the hippocampus, neocortex, septum, amygdala, and hypothalamus [37,38,39]. Gepirone acts as a full agonist at 5-HT_1A_ autoreceptors and as a partial agonist at postsynaptic 5-HT_1A_ receptors [40]. Radioligand-binding studies indicate a high affinity for 5-HT_1A_ receptors (Ki ≈ 31.8 nM), whereas its affinity for 5-HT_2A_ receptors is substantially lower (Ki ≈ 3630 nM), underscoring its strong selectivity for the 5-HT_1A_ subtype [41]. The functional effects of gepirone on serotonergic transmission are time-dependent. Short-term administration suppresses serotonergic firing through activation of 5-HT_1A_ autoreceptors, whereas chronic treatment induces desensitization of these autoreceptors, ultimately enhancing 5-HT release in projection areas [33]. A similar effect arises from agonistic action at postsynaptic 5-HT_1A_ receptors [42] (Figure 3). This selective mechanism distinguishes gepirone from commonly prescribed antidepressants such as SSRIs and SNRIs. The dual action of gepirone at both pre- and postsynaptic 5-HT_1_A receptors contributes to its antidepressant and anxiolytic efficacy while simultaneously reducing the risk of side effects frequently associated with SSRIs, such as nausea and sexual dysfunction [43,44].

Unlike benzodiazepines, gepirone does not act on the GABA-A/benzodiazepine receptor complex. Nevertheless, it produces anxiolytic effects, likely through its partial agonism at 5-HT_1A_ receptors [45]. Its selectivity for 5-HT1A receptors over D2 receptors is a defining characteristic, and this selectivity is greater than that of buspirone, tandospirone, and perospirone (other azapirones with anxiolytic properties), making gepirone potentially more effective and better tolerated [42].

The clinical efficacy of gepirone also reflects the activity of its two principal metabolites: 1-(2-pyrimidinyl)-piperazine (1-PP) and 3′-hydroxy-gepirone (3′-OH) [46] (Figure 2). The latter demonstrates significant pharmacological activity as a 5-HT_1_A receptor agonist (Ki = 58 nM) with proven antidepressant and anxiolytic effects [17,20]. In contrast, 1-PP does not bind directly to 5-HT receptors but functions as an antagonist at presynaptic α_2_-adrenoreceptors (Ki = 42 nM), a mechanism that may further support antidepressant efficacy [20].

### 2.2. Pharmacokinetics

The initial clinical investigations with the immediate-release (IR) formulation of gepirone confirmed its antidepressant potential, but its therapeutic value was limited by pharmacokinetic drawbacks. Owing to its short half-life, frequent dosing was required, and the resulting high plasma peaks hindered dose escalation due to tolerability issues [18,33]. To address these challenges, gepirone was subsequently reformulated as an extended-release (ER) tablet. Administered once daily, the ER formulation ensures gradual and sustained gastrointestinal absorption, reducing the pronounced peak–to-trough fluctuations typical of IR dosing. This smoother pharmacokinetic profile is associated with improved tolerability, fewer adverse effects, and greater adherence, while also allowing for higher, more effective dosing. In light of these advantages, only the ER formulation has received regulatory approval for the treatment of depressive disorders. Therefore, in the remainder of this review, we will focus more specifically on gepirone ER. To better understand the superiority of ER formulations over IR, Table 1 presents the key differences between them, particularly those relevant to clinical efficacy and tolerability.

#### 2.2.1. Absorption

The absolute bioavailability of gepirone is estimated at 14–17% [40]. Following oral administration of the extended-release formulation (EXXUA^®^), the maximal plasma concentration (Cmax = 3.6–4.3 ng/mL) is typically reached within 6 h post-dose (Tmax) [18] (Table 2). A steady-state plasma concentration is achieved after 4 days of daily dosing [22]. Gepirone administered in the IR form is absorbed throughout the entire length of the small intestine without a specific absorption window, even under simulated extended-release conditions [47]. In contrast, gepirone-ER is absorbed to a greater extent in the distal parts of the small intestine, which leads to reduced production of the metabolite 1-PP compared to the IR formulation [18].

Food intake significantly affects the pharmacokinetic parameters of EXXUA^®^, due to higher hepatic blood flow and reduced metabolism of this drug [46]. Both Cmax and Tmax are diet-dependent. After a high-fat meal, Tmax is shortened to approximately 3 h. Compared with the fasted state, plasma Cmax increases by 27% after a low-fat meal (~200 kcal), by 55% after a medium-fat meal (~500 kcal), and by 62% after a high-fat meal (~850 kcal). The impact of diet on overall exposure (AUC) is less pronounced than on Cmax. Specifically, AUC increases by 14% after a low-fat meal, 22% after a medium-fat meal, and 32–37% after a high-fat meal [46]. Similarly, systemic exposure to the major metabolites 3′-OH-gepirone and 1-PP is consistently higher under fed conditions, and the magnitude of the food effect parallels that observed for gepirone itself [48]. High-fat meals can inhibit epithelial efflux transporters (e.g., permeability-glycoprotein; P-gp), thereby decreasing the transport of drugs with a high extraction ratio (such as geprione), which leads to an increase in their bioavailability [46,49]. Moreover, EXXUA, as a water-soluble drug, may be less absorbed in the gastric tract due to fat-meal–induced bile secretion and micelle formation. Hydrophilic drugs can become trapped inside amphiphilic micelles, resulting in delayed absorption and a reduced free drug fraction [31,49]. In contrast, Gepirone IR intake after high-fat meal influenced only AUC (about 40% higher) and Tmax (increase to 2 h) of while Cmax was not affected [50].

#### 2.2.2. Distribution

The lipophilic nature of gepirone contributes to its wide tissue distribution. Animal studies have demonstrated accumulation in the brain, lungs, kidneys, and adipose tissue [40]. The apparent volume of distribution for the extended-release formulation is estimated at 94.5 L [31]. Importantly, the drug rapidly crosses the blood–brain barrier, enabling central nervous system activity [51].

In vitro studies show that plasma protein binding of gepirone is independent of concentration and accounts for 72% of the parent compound, 59% for its active metabolite 3′-OH-gepirone, and 42% for 1-PP [31]. Gepirone does not significantly alter the plasma protein binding of other antidepressant or antipsychotic agents. Owing to its relatively low binding affinity, it is unlikely to participate in clinically relevant drug–drug interactions associated with displacement from protein-binding sites, such as those observed with highly bound drugs like warfarin [31].

#### 2.2.3. Metabolism

The half-life of gepirone is reported by the FDA to be approximately 5 h (Fabre-Kramer Pharmaceuticals Inc., Gepirone ER). In a pharmacokinetic study evaluating ER-gepirone, the half-life was determined to be 6.1 ± 3.7 h after administration of 20 mg daily for 2 days, followed by 40 mg daily for 5 days in seven healthy subjects under fasting conditions [22].

Gepirone undergoes extensive hepatic metabolism, primarily mediated by cytochrome P450 enzymes. Cytochrome P450 3A4 (CYP3A4) is the main isoenzyme responsible for the biotransformation of gepirone into its major metabolites, including 1-(2-pyrimidinyl)-piperazine (1-PP) and 3′-hydroxy-gepirone (3′-OH-gepirone) (both exceed the concentrations of the parent compound and contribute to the overall clinical efficacy of gepirone; Figure 2), and other minor metabolites. In addition, cytochrome P450 2D6 (CYP2D6) contributes to gepirone metabolism, although it does not participate in the formation of 1-PP [47,52].

#### 2.2.4. Excretion

Gepirone is rapidly eliminated from the human body, with approximately 60% of the administered dose excreted within the first 24 h [31]. The mean elimination half-life (T1/2) is estimated at 2.9 h for gepirone and 5.8 h for its metabolite 1-PP [50].

Excretion occurs predominantly via the renal route. Following administration of a single [14C]-labeled oral dose, about 81% of the radioactivity was recovered in the urine and 13% in the feces, mainly in the form of metabolites [40]. Importantly, elimination is not significantly altered in patients with hepatic or renal impairment, indicating that apparent clearance of EXXUA^®^ remains stable in these populations [48].

## 3. Preclinical Development of Gepirone

Gepirone, a selective 5-HT_1A_ receptor agonist, has been widely investigated for its potential anti-agressive, anxiolytic- and antidepressant-like properties using animal models (Table 2), which provided a starting point for further evaluation of its therapeutic effects in clinical trials.

### 3.1. Electrophysiological Studies

The pharmacological profile of gepirone has been extensively characterized through neurophysiological and behavioral studies, establishing it as a prototypical selective 5-HT_1A_ receptor agonist. Early investigations by McMillen et al. [53] demonstrated profound effects of gepirone on dopaminergic and serotonergic systems in rodents. In Sprague–Dawley rats, acute intravenous administration (2.3–10 mg/kg) markedly inhibited the firing activity of dopaminergic neurons in the substantia nigra pars compacta. Interestingly, apomorphine (D_2_ receptor agonist) administered after gepirone did not alter this inhibitory response, whereas pretreatment with apomorphine reduced neuronal activity that was further suppressed by gepirone. Haloperidol (D_2_ receptor antagonist) reversed the inhibitory effect only in the context of apomorphine co-administration, but not when apomorphine preceded gepirone, suggesting that dopaminergic pathways play only a minor role in mediating gepirone’s effects [53]. By contrast, serotonergic neurons in the dorsal raphe nuclei were considerably more sensitive to gepirone. Cumulative doses as low as 0.008 mg/kg reduced firing rates by nearly 90% (88.3 ± 5.1%), an effect only partially reversed by 5-HT antagonists. The partial reversal, together with the observation that mCPP (5-HT receptors agonist) was less effective than gepirone, underscored the drug’s strong affinity for 5-HT1A receptors, particularly postsynaptic sites, and comparatively weak affinity for D receptors. Gepirone’s metabolite, 1-PP, further supported this conclusion: although it increased neuronal firing at low doses (1–5 mg/kg), higher doses (10 mg/kg) induced depolarization block, which was reversed by apomorphine [53].

Long-term studies provided additional mechanistic insights. Blier and de Montigny [54] found that repeated gepirone administration (15 mg/kg/day) suppressed spontaneous and evoked activity of dorsal raphe 5-HT neurons. This suppression gradually normalized after 7–14 days, with full recovery at two weeks, a time course consistent with desensitization of somatodendritic autoreceptors and sustained stimulation of postsynaptic 5-HT_1A_ receptors. Chronic gepirone also attenuated neuronal responsiveness not only to 5-HT itself but also to LSD (5-HT autoreceptor agonist), 8-OH-DPAT (5-HT_1A_ receptor full agonist), and gepirone, whereas GABAergic responses remained unaffected. These data strongly support the hypothesis that gepirone reduces serotonergic activity through feedback desensitization of autoreceptors, while postsynaptic 5-HT_1A_ receptor stimulation persists as a central mechanism. Notably, gepirone exerted stronger effects on raphe serotonergic neurons compared with hippocampal pyramidal neurons, without disrupting ascending serotonergic projections, further highlighting its selective modulatory properties [54].

### 3.2. Behavioral Studies

#### 3.2.1. Anti-Aggressive Properties of Gepirone

The neurophysiological evidence was paralleled by robust behavioral findings. McMillen et al. [55] investigated gepirone’s anti-aggressive profile in CD-1 mice exposed to isolation-induced aggression. Acute intraperitoneal administration (1.25–10 mg/kg) produced a dose-dependent reduction in aggressive behavior, with greater potency than mCPP or TFMPP (5-HT receptor agonists). The anti-aggressive effects of gepirone (5 mg/kg) were potentiated by methiothepin (5-HT_1_ and 5-HT_2_ antagonist) and methysergide (5-HT antagonist), suggesting a serotonergic mechanism involving postsynaptic 5-HT_1A_ receptor stimulation and reduced serotonergic tone. By contrast, the metabolite 1-PP was considerably less effective, producing only partial inhibition at higher doses. Interestingly, inhibition of gepirone metabolism with SKF 525A (cytochrome P450 inhibitor; 20 mg/kg) enhanced its efficacy, completely abolishing aggression when co-administered with gepirone (5 mg/kg). Biochemical assays aligned with these behavioral findings. Gepirone modified striatal dopamine metabolism, reducing homovanillic acid (HVA) at low doses (1.25–2.5 mg/kg) but increasing it at 5 mg/kg. DOPAC concentrations were elevated at both 2.5 and 5 mg/kg, whereas dopamine metabolites remained unchanged in the prefrontal cortex. Importantly, gepirone reduced 5-HIAA levels in both striatum and prefrontal cortex, strongly implicating reduced 5-HT turnover in its anti-aggressive actions. Binding assays confirmed high affinity for hippocampal 5-HT_1A_ receptors, modest affinity for 5-HT_2_ receptors, and negligible binding to D_2_ receptors, highlighting gepirone’s pharmacological specificity [55].

These results were replicated and extended in subsequent models. Lopez-Mendoza et al. [56] demonstrated that gepirone (2.5–10 mg/kg i.p.) significantly increased attack latency and suppressed territorial aggression in BALB/C mice, with near-complete inhibition at 10 mg/kg. Crucially, these effects were blocked by (+)WAY 100135, a 5-HT_1A_ antagonist, and were not accompanied by sedation or motor impairment, indicating selective anxiolytic and anti-aggressive activity. A follow-up study using the more selective antagonist WAY 100635 confirmed these results, while also showing anxiolytic-like effects of gepirone (7.5 mg/kg) in the elevated plus maze [56].

Further validation was provided in testosterone-induced aggression models in Fischer rats [57]. Gepirone (1–10 mg/kg i.p.) reduced aggressive behavior with potency comparable to buspirone and 8-OH-DPAT, and greater than drugs acting on 5-HT_1B_/5-HT_2C_ receptors (eltoprazine, TFMPP, DOM) or non-serotonergic compounds (e.g., chlordiazepoxide, morphine). The anti-dominant effects of gepirone were reversed by serotonergic antagonists such as pizotifen, pirenperone, and pindolol, further implicating serotonergic pathways. Chronic treatment selectively reduced 5-HT and 5-HIAA levels in the hippocampus, but not in the striatum or frontal cortex, strongly suggesting that hippocampal 5-HT_1A_ receptors mediate gepirone’s anti-aggressive actions [57].

#### 3.2.2. Anxiolytic-like Properties of Gepirone

In parallel with its anti-aggressive profile, gepirone demonstrated consistent anxiolytic properties across multiple preclinical paradigms. Costello et al. [58] assessed gepirone in Long-Evans rats using a conditioned suppression of drinking paradigm under varying shock predictability. Gepirone (1.25–5 mg/kg i.p.) increased licking behavior under predictable and moderately predictable shock conditions, effects indicative of reduced anxiety. Notably, non-shock-related licking remained unaffected, suggesting specificity for anxiety-related responses. However, under unpredictable conditions, gepirone reduced licking at all doses, with only the lowest dose recovering to baseline—an outcome interpreted as a proconflict effect at higher doses in unpredictable aversive contexts [58]. Yamashita et al. [59] corroborated these findings in Wistar rats, showing that both acute (1–10 mg/kg i.p.) and repeated (5–10 mg/kg i.p. for 7 days) gepirone significantly increased shock-suppressed licking. Repeated treatment produced even greater effects than buspirone, with nearly six-fold increases at 5 mg/kg. Importantly, neither drug altered nociceptive thresholds or baseline drinking, confirming anxiolytic specificity [59]. Mechanistic studies with serotonergic lesions induced by 5,7-dihydroxytryptamine (5,7-DHT) revealed that gepirone’s effects required intact serotonergic pathways. In intact rats, oral gepirone (10 mg/kg) enhanced licking nearly forty-fold, while this effect was blunted in lesioned animals. Gepirone also induced serotonin-syndrome-like behaviors across a broad dose range, particularly in intact animals, consistent with its role as a direct 5-HT_1A_ agonist [60]. Other behavioral assays provided convergent evidence. In the potentiated startle test, gepirone (5–10 mg/kg) and buspirone (1.25–5 mg/kg) attenuated startle amplitude, with early transient increases at lower doses—a pattern not seen with benzodiazepines [61]. Söderpalm et al. [62] employed the Montgomery Conflict Test in Sprague–Dawley rats and showed that gepirone (32–128 nmol/kg s.c.) increased open-arm entries and time spent in open arms, while very high doses impaired locomotion. Buspirone displayed a similar profile, whereas ipsapirone was effective at higher doses. Comparative agents such as PMP and 8-OH-DPAT produced either anxiogenic or biphasic effects, reinforcing gepirone’s unique pharmacological signature [62].

The importance of treatment duration was emphasized by Motta et al. [63], who found that acute gepirone (1–10 mg/kg i.p.) sometimes produced anxiogenic-like responses in Wistar rats, including reduced open-arm exploration, whereas chronic treatment (10 mg/kg/day p.o. for 14 days) in socially isolated animals increased open-arm entries and time spent in open arms [63]. This distinction was replicated in elevated plus-maze studies, where acute gepirone increased grooming and reduced exploratory behavior, while chronic treatment reversed these effects, producing robust anxiolytic outcomes [64]. Notably, fluoxetine produced weaker responses, suggesting gepirone’s superior efficacy after chronic dosing [64].

The novelty-suppressed feeding paradigm provided further evidence. Acute gepirone (4 mg/kg i.p.) did not alter feeding latency in Long-Evans rats, whereas 21 days of treatment significantly reduced latency, mirroring buspirone and mianserin [65]. Unlike SSRIs, which were anxiogenic when given acutely but anxiolytic after chronic treatment, gepirone did not provoke acute anxiogenesis, highlighting a more favorable therapeutic profile. In addition, gepirone suppressed ultrasonic distress vocalizations in Wistar rats in a dose-dependent manner (0.3–10 mg/kg i.p. or 1–30 mg/kg p.o.), achieving near-complete inhibition at higher doses. Gepirone was more potent than buspirone and comparable to benzodiazepines, while 8-OH-DPAT remained the most effective [66].

#### 3.2.3. Antidepressant-like Properties of Gepirone

The 5-HT_1A_ receptor plays a pivotal role in mood regulation, and its pharmacological modulation has been strongly implicated in the pathophysiology and treatment of depression. Given gepirone’s anxiolytic efficacy, selective receptor binding profile, and pharmacological similarity to buspirone, several studies have investigated its antidepressant potential in preclinical models, both as a stand-alone treatment and in combination with other agents.

One of the earliest investigations was conducted by Przegaliński et al. [67], who evaluated gepirone in the forced swimming test (FST, or Porsolt test) in Wistar rats. Gepirone (2.5–20 mg/kg i.p.) reduced immobility time in a dose-dependent manner, with acute administration of 20 mg/kg producing the most robust effect. Importantly, its efficacy was greater than that of buspirone or ipsapirone, neither of which produced significant reductions in immobility under acute dosing conditions. Gepirone was also effective after repeated administration, unlike buspirone and ipsapirone, except for the latter at 5 mg/kg. Profiden, a metabolic inhibitor, potentiated the effects of all three drugs, but this enhancement was blocked by their common metabolite, 1-PP, implicating both parent compound and metabolites in the overall effect. Open-field testing revealed no locomotor impairment at effective doses, except for a mild reduction in activity at the highest dose of 20 mg/kg. The authors concluded that gepirone’s antidepressant-like effect could not be fully explained by 1-PP, since ipsapirone generated lower levels of this metabolite yet lacked significant efficacy [67]. These findings were reinforced by Chojnacka-Wójcik et al. [68], who confirmed that acute gepirone administration (2.5–20 mg/kg i.p.) produced a clear dose–response reduction in immobility in the FST. Antagonist studies revealed mechanistic insights: NAN-190 (5-HT_1A_/α_1_ antagonist), pindolol (β-blocker with 5-HT_1A_ activity), spiperone (5-HT_1A_/5-HT_2_/D_2_ antagonist), and haloperidol (D_2_ antagonist) attenuated gepirone’s effect, implicating primarily postsynaptic 5-HT_1A_ receptors with additional contributions from dopaminergic systems. Strikingly, serotonin-depleting agents such as p-chloroamphetamine and p-chlorophenylalanine—reducing 5-HT and 5-HIAA by over 80%—did not abolish gepirone’s activity, further supporting a postsynaptic mechanism largely independent of 5-HT availability [68]. Comparative studies by Benvenga and Leander [69] evaluated gepirone (5–20 mg/kg i.p.) against imipramine (tricyclic antidepressant), 8-OH-DPAT, and LY228729 (5-HT_1A_ receptor agonist) in the FST. Gepirone was effective only at 20 mg/kg but did not impair locomotion, whereas imipramine reduced immobility but caused sedation. LY228729 showed antidepressant-like activity but produced transient adverse effects such as flat body posture. These results highlighted gepirone’s favorable profile, producing antidepressant-like effects without confounding motor impairment [69].

The learned helplessness (LH) model provided additional insights. Giral et al. [70] reported that repeated gepirone (0.06–0.125 mg/kg i.p.) reduced escape failures, particularly at 0.125 mg/kg, with efficacy comparable to ipsapirone and somewhat lower than buspirone or 8-OH-DPAT. In contrast, Drugan et al. [71] observed no significant effect of gepirone (2–5 mg/kg i.p.) on escape latency in Sprague–Dawley rats, attributing this to conservative dosing aimed at avoiding serotonergic adverse effects. Later, Camargo et al. [72] examined the influence of early-life nutrition on gepirone’s efficacy in the LH paradigm. Gepirone (2.5–7.5 mg/kg i.p.) improved escape performance in malnourished and control rats exposed to inescapable stress, though some effects extended to non-shocked controls. Variability across studies emphasized the narrow effective dose range and sensitivity of gepirone’s actions to experimental conditions [72].

Beyond rodent models, Barrett et al. [73] tested gepirone in White Carneaux pigeons under punished responding paradigms. Gepirone (0.03–10 mg/kg i.m.) increased responding under fixed-interval schedules at low-to-moderate doses, whereas buspirone consistently suppressed responding. These results suggested that gepirone acts predominantly via serotonergic pathways, in contrast to buspirone, whose mixed serotonergic and dopaminergic actions may contribute to different behavioral outcomes.

**Table 2:** Summary of preclinical studies on gepirone: Animal models and receptor activity.

Study	Species/Strain	Model/Treatment	Findings
McMillen et al. (1987) [53]	Male Sprague-Dawley rats	In vivo recording of single cell impulse flow–dopamine cells N = 6/4 Acute: ‐ Gepirone (2.3–10 mg/kg i.v.) ‐ Apomorphine (5–20 μg/kg/20 mg/kg i.v.) ‐ Haloperidol (0.03 or 0.5 mg/kg i.v.) ‐ 1-PP (0.8–16 mg/kg i.v.)	Gepirone alone: ↓Firing rate Gepirone (2 × 10 mg/kg) → Apomorphine → haloperidol (0.5 mg/kg): ↓Firing rate after gepirone ↔Firing rate after apomorphine ↑Firing rate after haloperidol Apomorphine → Gepirone (2 × 2.3 mg/kg) → haloperidol (0.5 mg/kg): ↓Firing rate after apomorphine ↔Firing rate after gepirone ↔Firing rate after haloperidol 1-PP alone: ↑Firing rate (0.8–5 mg/kg) ↑Firing rate led to depolarization block (10 mg/kg) ↓Depolarization block by apomorphine (10–20 μg/kg) Apomorphine (5.0–7.5 μg/kg) → 1-PP (16 mg/kg)→ Haloperidol (0.03 mg/kg): ↔Firing rate after apomorphine + 1-PP ↑Firing rate after haloperidol
		In vivo recording of single cell impulse flow–serotonin cells N = 8 Acute: ‐ Gepirone (0.005-0.1 mg/kg i.v.) ‐ Methiotepin (0.5 mg/kg i.v.) ‐ Methysergide (0.16 mg/kg i.v.) ‐ mCPP (0.01-0.04 mg/kg i.v.)	Gepirone alone: ↓Firing rate Gepirone (0.008 mg/kg) → Methiothepin: ↓Firing rate after gepirone ↔Firing rate (partial) after methiothepin Gepirone (0.016 mg/kg) + methysergide: ↓Firing rate after gepirone; ↔Firing rate (patrial) after methysergide mCPP alone: ↓Firing rate (less effective than gepirone)
McMillen et al. (1987) [53]	Male Sprague-Dawley rats	Affinity for dopamine and serotonin receptors (IC_50_) N = 3–4 per group Acute (various drugs conc.): ‐ Gepirone ‐ Buspirone ‐ mCPP ‐ TFMPP ‐ 8-OH-DPAT ‐ Methysergide ‐ Methiotepin	Affinity to 5HT_1_ receptors in the frontal cortex: ↑Gepirone, Buspirone, mCPP, TFMPP, 8-OH-DPAT, Methysergide, Mthiothepin Affinity to 5HT_1_ receptors in hippocampus: ↑Gepirone, Buspirone, TFMPP, 8-OH-DPAT, Methysergide, Methiothepin ↔mCPP Affinity to 5HT_2_ receptors in frontal cortex: ↑8-OH-DPAT, Methysergide, Methiothepin ↔mCPP, TFMPP ↓Gepirone, Buspirone Affinity to D_2_ receptors in striatum: ↑mCPP, 8-OH-DPAT, Methiothepin ↔Buspirone ↓Gepirone, TFMPP, Methysergide
Blier and de Montigny (1987) [54]	Male Sprague-Dawley rats	In vivo single cell recording N = 3 2/7/14 days and 14 days + 48 h recording Gepirone (10 mg/kg, 15 mg/kg; s.c.) LSD (2.5 μg/kg–25 μg/kg, i.v.) GABA 8-OH-DPAT (2.5 μg/kg i.v.)	Gepirone alone: 2 days; 10 mg/kg per day: ↔Spontaneously active 5-HT neurons ↓Firing frequency of 5-HT neurons 2 days; 15 mg/kg per day: ↓Spontaneously active 5-HT neurons ↓Firing frequency of 5-HT neurons 7 days; 15 mg/kg per day: ↔Spontaneously active 5-HT neurons ↓Firing frequency of 5-HT neurons (partially recovered) 14 days; 15 mg/kg per day: ↔Spontaneously active 5-HT neurons ↔Firing frequency of 5-HT neurons (partially recovered) 14 days + 48 h/15 mg/kg: ↔Firing rate Gepirone (14 days/15 mg/kg) + LSD: ↑ED50 Gepirone (14 days + 48 h/15 mg/kg) + LSD: ↑ED50 Gepirone 14 days/15 mg/kg per day: ↓Responsivenes of 5HT neurons to 5-HT, LSD, 8-OH-DPAT and gepirone ↔Responsivenes of 5HT neurons to GABA ↔Firing activity by 8-OH-DPAT ↓Firing activity by gepirone and 8-OH-DPAT in CA1 and CA3 pyramidal neurons. Effectiveness in dorsal hippocamus and dorsal raphne: ↑Effectiveness in raphne 5HT neurons than hippocampal pyramidal neurons (gepirone and 8-OH-DPAT ↑Effectiveness in hippocampus than in raphne (5-HT) ↔Effectiveness after gepirone treatment for 14 days/15 mg/kg per day Geprione effect on synaptic transmission: ↔Synaptic transmission (acute/10–50 μg/kg) ↔Synaptic transmission (14 days/15 mg/kg per day)
McMillen et al. (1989) [55]	Male CD-1 mice	Intraspecies aggression induced by isolation N = 5–8 per group Gepirone/TFMPP (i.p.): ‐ 1.25 mg/kg; ‐ 2.5 mg/kg; ‐ 5 mg/kg; ‐ 10 mg/kg; 8-OH-DPAT (i.p.): ‐ 0.25 mg/kg; ‐ 0.5 mg/kg; ‐ 1 mg/kg; ‐ 2 mg/kg; mCPP (i.p.): ‐ 1 mg/kg; ‐ 3 mg/kg; ‐ 5 mg/kg; ‐ 10 mg/kg; Methysergide (2.5 mg/kg i.p.) Methioithepin (0.25 mg/kg i.p.) SKF 525A (20 mg/kg i.p.) 1-PP (10 mg/kg i.p.)	Gepirone alone: ↓Number of fighting (dose-dependent; 1/8 at 10 mg/kg) ↔Time on rotating rod (5–10 mg/kg) mCPP alone: ↓Number of fighting (dose-dependent; 1/8 at 10 mg/kg) ↓Time on rotating rod (5–10 mg/kg) TFMPP alone: ↓Number of fighting (1/8 at 5 mg/kg; total inhibition at 10 mg/kg) ↓Time on rotating rod (similar to mCPP) 8-OH-DPAT alone: ↓Number of fighting (dose-dependent; total inhibition at 2 mg/kg) ↔Time on rotating rod (1 mg/kg) Gepirone + Methiothepin/Methysergide: ↓Number of fighting (more potent; total inhibition at 5 mg/kg) ↔Time on rotating rod (Methysergide; Gepirone at 5 mg/kg) ↓Time on rotating rod (Methiothepin; Gepirone at 5 mg/kg) Methiothepin/Methysergide alone: ↔Number of fighting ↔Time on rotating rod mCPP + Methiothepin/Methysergide: ↓Number of fighting (no change from mCPP alone) ↔Time on rotating rod (Methysergide; mCPP at 5 mg/kg) ↓Time on rotating rod (Methiothepin; mCPP at 5 mg/kg) 8-OH-DPAT + Methysergide: ↓Number of fighting (more potent; total inhibition at 1 mg/kg) ↔Time on rotating rod (Methysergide; 8-OH-DPAT at 1 mg/kg) ↓Time on rotating rod (Methiothepin; 8-OH-DPAT at 1 mg/kg) 1-PP alone: ↓Number of fighting (4/8 at 10 mg/kg) SKF 525A + Gepirone (5 mg/kg): ↓Number of fighting (more potent; total inhibition)
McMillen et al. (1987) [55]	Male Sprague-Dawley rats	Monoamine metabolism (DOPAC/HVA/5HIAA) N = 6–9 per group Gepirone (i.p.) ‐ 0.5 mg/kg; ‐ 1.25 mg/kg; ‐ 2.5 mg/kg; ‐ 5 mg/kg.	Gepirone-Striatum: ↔HVA conc. (0.5 mg/kg) ↓HVA conc. (1.25–2.5 mg/kg) ↑HVA conc. (5 mg/kg) ↔DOPAC conc. (0.5–1.25 mg/kg) ↑DOPAC conc. (2.5–5 mg/kg) ↔5HIAA conc. (0.5–1.25 mg/kg) ↓5HIAA conc. (2.5–5 mg/kg) Gepirone–prefrontal cortex: ↔HVA conc. ↔DOPAC conc. ↔5HIAA conc.
Lopez-Mendoza et al. (1998) [56]	Male BALB/C mice	Territorial aggression inducted by isolation N = 48 Gepirone (i.p.): ‐ 1 mg/kg ‐ 2.5 mg/kg; ‐ 5 mg/kg; ‐ 7.5 mg/kg. WAY 100635 (i.p.): ‐ 1.5 mg/kg; ‐ 2.5 mg/kg; ‐ 5 mg/kg.	Gepirone alone: ↓ Latency of attack ↓Attack frequency ↓Tail rattling ↔Grooming ↔Motor impairment ↑Exploratory sniffing ↑Social sniffing (2.5 and 7.5 mg/kg) WAY 100653 alone: ↔ Latency of attack ↔Attack frequency ↔Tail rattlin ↔Motor impairment ↔Exploratory sniffing ↔Social sniffing ↑Grooming Gepirone + WAY 100653: ↓ Latency of attack ↑Attack frequency ↑Tail rattling ↔Grooming ↔Motor impairment ↔Exploratory sniffing ↔Social sniffing
Lopez-Mendoza et al. (1998) [56]	Male BALB/C mice	Elevated plus-maze N= 48 Gepirone (i.p.): ‐ 1 mg/kg ‐ 2.5 mg/kg; ‐ 5 mg/kg; ‐ 7.5 mg/kg. WAY 100635 (i.p.): ‐ 1.5 mg/kg; ‐ 2.5 mg/kg; ‐ 5 mg/kg.	Gepirone alone: ↔Open and closed arms entries ↑Time spent in open arms (7.5 mg/kg) ↓Protected head dips (7.5 mg/kg) ↓Protected stretches (7.5 mg/kg) ↓Returning into closed arms from center platform WAY 100635 alone: ↔Open and closed arms entries ↑Time spent in open arms (5 mg/kg) ↓Protected head dips (5 mg/kg) ↔Protected stretches ↔Returning into closed arms from center platform Gepirone + WAY 100635: ↓Open and closed arms entries (7.5 mg/kg Gepirone vs. 7.5 mg/kg Gepirone + WAY 100635) ↓Time spent in open arms (compared to Gepirone alone) ↑ Protected head dips (7.5 mg/kg Gepirone vs. 7.5 mg/kg Gepirone + WAY 100635) ↓ Protected head dips (2.5 mg/kg Gepirone + 5 mg/kg WAY 100635) ↑ Protected stretches (7.5 mg/kg Gepirone vs. 7.5 mg/kg Gepirone + WAY 100635) ↔Returning into closed arms from center platform–exception: ↑Returning (7.5 mg/kg Gepirone vs. 7.5 mg/kg Gepirone + 1.5 mg/kg WAY 100635)
Bonson et al. (1994) [57]	Male Fischer rats	Androgen-induced dominance N = 344 Testosterone–30 mg/kg s.c./14 days Gepirone/buspirone/8-OH-DPAT (1–10 mg/kg i.p.) Eltoprazine (1, 3 and 7 mg/kg i.p.) TFMPP (0–8 mg/kg i.p.) DOM (0, 1, 3, 6 mg/kg) Chlordiazepoxide (3, 10 and 20 mg/kg i.p.) Morphine (3 and 6 mg/kg i.p.) Pizotyline (10 mg/kg i.p.) Pirenperone (0.16 mg/kg i.p.) Pindolol (5 mg/kg i.p.)	Gepirone/Buspirone/8-OH-DPAT: ↓Dominant effect (in testosterone-treated rats)-dose dependent ↑Flattened body posture (10 mg/kg) ↔Dominant effect (in non-testosterone treted rats) ↑ Dominant effect (Gepirone 10 mg/kg; pretreated with Pizotyline/Pirenperone/Pindolol and 8-OH-DPAT 1 mg/kg pretreated with Pindolol) ↔Dominant effect (Buspirone 10 mg/kg pretreated with Pizotyline/Pirenperone/Pindolol and 8-OH-DPAT 1 mg/kg pretreated with Pizotyline/Pirenperone) Eltroprazine: ↓Dominant effect (in Testosterone treated rats)–dose dependent ↔Dominant effect (in non-testosterone treated rats) ↔Dominant effect (6 mg/kg pretreated with Pizotyline/Pirenperone/Pindolol) TFMPP: ↓Dominant effect (in Testosterone treated rats) ↑Hindleg abduction (8 mg/kg) ↑Purposless chewing (8 mg/kg) ↑Hallucinogenics pause (8 mg/kg) ↔Dominant effect (in non-testosterone treated rats) DOM: ↓Dominant effect (in Testosterone treated rats) ↑Head shakes and contraction of back muscles (6 mg/kg) ↑Purposless chewing (6 mg/kg) ↑Hallucinogenics pause (6 mg/kg) ↔Dominant effect (in non-testosterone treated rats) Chlordiazepoxide: ↔Dominant effect (in Testosterone treated rats at 3 and 10 mg/kg) ↓Dominant effect (in Testosterone treated rats at 20 mg/kg)–lack of motor ability to move ↔Dominant effect (in non-testosterone treated rats) Morphine: ↓Dominant effect (in Testosterone treated rats at 6 mg/kg) ↔Dominant effect (in non-testosterone treated rats) ↔Dominant effect (6 mg/kg pretreated with Pizotyline/Pindolol) ↑Dominant effect (6 mg/kg pretreated with Pirenperone)
Costello et al. (1991) [58]	Female Long-Evans rats	Conditioned suppression of drinking n = 9–10 per group repeated (5 days) treatment Gepirone (i.p.): ‐ 1.35 mg/kg; ‐ 2.5 mg/kg; ‐ 5 mg/kg.	Predictable schedule: Gepirone (1.25–2.5 mg/kg): ↑Water Liking on day 4 ↔Water licking on days 1–3 and 5 Gepirone (5 mg/kg): ↑Water licking on day 5 ↔Water licking on days 1–4 Moderate predictible schedule: Gepirone (1.25 mg/kg): ↑Water licking on days 4–5 ↔Water licking on days 1–3 Gepirone (2.5 mg/kg): ↔Water licking on days 1–5 Gepirone (5 mg/kg): ↔Water licking on days 3–5 ↓Water licking on days 1–2 Unpredictible schedule: Geprirone (1.25 mg/kg) ↔Water licking on days 2–5 ↓Water licking on day 1 Gepirone (2.5–5 mg/kg) ↓Water licking on days 1–5
Yamashita et al. (1995) [59]	Male Wistar rats	Drinking conflict test N = 3–5 per group Acute/repeated (7 days) treatment Gepirone (i.p.): ‐ 1 mg/kg; ‐ 2 mg/kg; ‐ 5 mg/kg; ‐ 10 mg/kg. Buspirone (i.p.): ‐ 5 mg/kg; ‐ 10 mg/kg.	Acute treatment: Gepirone (1–10 mg/kg): ↑Water licking Buspirone (5 and 10 mg/kg): ↑Water licking Buspirone (5–10 mg/kg) < Gepirone (2–10 mg/kg) 2. Chronic treatment: Gepirone (5 and 10 mg/kg): ↑Water licking Buspirone (5 and 10 mg/kg): ↑Water licking Buspirone (5–10 mg/kg) < Gepirone (5–10 mg/kg)
Eison et al. (1986) [60]	Male Sprague-Dawley rats	Drinking conflict test N = 8–10 Serotonin lesion by 5,7-DHT (150 μg/rat) Geprione, buspirone, diazepam: ‐ 10 mg/kg p.o.	Gepirone (non-lesioned rats): ↑Liking (38-fold) Buspirone (non-lesioned rats): ↑Licking (5-fold) Diazepam (non-lesioned rats): ↑Liking (19-fold) Gepirone (lesioned rats): ↔Licking (compared to control) Buspirone (lesioned rats): ↔Licking (compared to control) Diazepam (lesioned rats): ↔Licking (compared to control)
Eison et al. (1986) [60]	Male Sprague-Dawley rats	Serotonin syndrome N = 8–10 per group Serotonin lesion by 5,7-DHT (150 μg/rat i.v.) Gepirone, buspirone, dizepam (non-lesioned rats): ‐ 10 mg/kg i.p.; ‐ 20 mg/kg i.p.; ‐ 50 mg/kg i.p.; ‐ 50 mg/kg p.o.; ‐ 100 mg/kg p.o.; ‐ 200 mg/kg p.o.; Gepirone, buspirone, diazepam (lesioned rats): ‐ 5 mg/kg i.p.; ‐ 10 mg/kg i.p.; ‐ 20 mg/kg i.p.; ‐ 20 mg/kg p.o.; ‐ 50 mg/kg p.o.; ‐ 100 mg/kg p.o.	Gepirone: Non-lesioned rats: ↑Tremor ↑Straub tail ↑Muscular hypertonus ↑Treading ↑Hindlimb abduction ↓Lateral head waving Lesioned rats (i.p. administration–18-times potent; p.o. administarion 54-times poten): ↑Tremor ↑Straub tail ↑Muscular hypertonus ↑Treading ↑Hindlimb abduction ↓Lateral head waving Buspirone and Diazepam: ↓Tremor ↓Straub tail ↓Muscular hypertonus ↓Treading ↓Hindlimb abduction ↓Lateral head waving
Eison et al. (1986) [60]	Male Sprague-Dawley rats	Acoustic startle test N = 8 per group Serotonin lesion by 5,7-DHT (150 μg/rat) Geprione, buspirone, diazepam– ‐ 1 mg/kg p.o.; ‐ 5 mg/kg p.o.; ‐ 10 mg/kg p.o.; ‐ 20 mg/kg p.o.	Non-lesioned rats: Gepirone: ↑Amplitude of the acustic startle reflex Buspirone: ↑Amplitude of the acustic startle reflex Diazepam: ↔Amplitude of the acustic startle reflex Lesioned rats: Gepirone: ↔Amplitude of the acustic startle reflex Buspirone: ↑Amplitude of the acustic startle reflex Diazepam: ↔Amplitude of the acustic startle reflex
Kehne et al. (1988) [61]	Male Sprague-Dawley rats	Potentiated startle testing N = 145 Gepirone (s.c.): ‐ 1.25 mg/kg; ‐ 2.5 mg/kg; ‐ 5 mg/kg; ‐ 10 mg/kg. Buspirone (s.c.): ‐ 0.6 mg/kg; ‐ 1.25 mg/kg; ‐ 2.5 mg/kg; ‐ 5 mg/kg; 1-PP (s.c.) ‐ 0.5 mg/kg; ‐ 20 mg/kg; ‐ 40 mg/kg.	Gepirone: ↔Startle amplitude in Noise-Alone trial ↓Startle amplitude in Light-Noise trial (5 and 10 mg/kg) Gepirone was less potent than Buspirone Buspirone: ↔Startle amplitude in Noise-Alone trial ↓ Startle amplitude in Light-Noise trial (1.25-5 mg/kg) 1-PP: ↔ Startle amplitude in Light-Noise trial
Söderpalm et al. (1989) [62]	Male Sprague-Dowley rats	Elevated lus maze test/Montgomery’s conflict test Gepirone (s.c.)/Buspirone (s.c)/Ipsapirone (s.c.)/PMP (s.c.): ‐ 8 nmol/kg; ‐ 32 nmol/kg; ‐ 128 nmol/kg; ‐ 512 nmol/kg; ‐ 2048 nmol/kg. 8-OH-DPAT (s.c.): ‐ 50 nmol/kg; ‐ 100 nmol/kg; ‐ 200 nmol/kg; ‐ 400 nmol/kg. L-5-HTP (i.p.): ‐ 28 μmol/kg; ‐ 56 μmol/kg; ‐ 112 μmol/kg; ‐ 224 μmol/kg; ‐ 448 μmol/kg. Benserazide (i.p.): 25 mg/kg.	Gepirone: ↑ Open arms entries (32 nmol/kg) ↑Time spent in open arms (32–128 nmol/kg) ↓Time spent in open arms (2048 nmol/kg) ↔Total number of entries (8–512 nmol/kg) ↓ Total number of entries (2048 nmol/kg) Buspirone: ↑ Open arms entries + time spent in open arms (32–128 nmol/kg) ↓ Open arms entries + time spent in open arms (2048 nmol/kg) ↑ Total number of entries (8 nmol/kg) ↔ Total number of entries (32–512 nmol/kg) ↓ Total number of entries (2048 nmol/kg) Ipsapirone: ↑ Open arms entries (32 nmol/kg) ↑Time spent in open arms (32–512 nmol/kg) PMP: ↓ Open arms entries time spent in open arms (32 and 512 nmol/kg) ↔ Total number of entries (8–512 nmol/kg) ↓ Total number of entries (2048 nmol/kg) 8-OH-DPAT: ↑ Open arms entries + time spent in open arms (100–200 nmol/kg) ↔Total number of entries (50–200 nmol/kg) ↓ Total number of entries (400 nmol/kg) L-5-HTP + Benserazide: ↑ Open arms entries (56 μmol/kg L-5-HTP) ↓ Open arms entries + time spent in open arms (448 μmol/kg L-5-HTP) ↔ Total number of entries (28–112 and 448 μmol/kg L-5-HTP) ↑ Total number of entries (224 μmol/kg L-5-HTP)
Motta et al. (1992) [63]	Male Wistar rats	Plus-maze test N = 6/8 per group Gepirone ‐ Acute (i.p.): ‐ 1 mg/kg; ‐ 3 mg/kg; ‐ 5.6 mg/kg; ‐ 10 mg/kg. Chronic (p.o.) ‐ 10 mg/kg. Ketanserin/prazosin (i.p.) ‐ 0.5 mg/kg; ‐ 1 mg/kg	Gepirone: Acute treatment: ↓Open arms entries (5.6 and 10 mg/kg) ↓Time spent in open arms (3–10 mg/kg) Acute treatment—group housed (14 days): ↓Open arms entries (10 mg/kg) ↓Time spent in open arms (10 mg/kg) Acute treatment—housed in isolation (14 days): ↔Open arms entries (10 mg/kg) ↔Time spent in open arms (10 mg/kg) Chronic treatment- housed in isolation (14 days): ↑Open arms entries; ↑Time spent in open arms. Katenserin: ↓Open arms entries (1 mg/kg) ↓Time spent in open arms Prazosin: ↔Open arms entries ↔Time spent in open arms
Silva and Brandão (2000) [64]	Male Wistar rats	Elevated Plus-maze Acute experiment (30 min before; n = 12 per group) gepirone (i.p.): ‐ 1 mg/kg; ‐ 3 mg/kg; ‐ 5.6 mg/kg; ‐ 10 mg/kg. Fluoxetine (i.p.): ‐ 5.6 mg/kg; ‐ 10 mg/kg. Chronic experiment (2 weeks treatment; n = 6 per group): Gepirone (p.o.): ‐ 10 mg/kg per day. Fluoxetine (p.o.) ‐ 10 mg/kg.	Acute treatment—Gepirone: ↑Grooming ↑Flat-back approach ↑Immobility ↔Open arms entries; ↔Center platform entries; ↔Enclose arms entries; ↔Peeping out ↔Sap ↓Time spent in open arms; ↓Rearing ↓Scanning ↓Head dipping ↓End arm activity Chronic treatment—Gepirone: ↑Open arms entries ↑Time spent in open arms ↑Time spent in close arms ↑Head dipping ↑End-arm activity ↔Peeping out ↔Grooming ↔Rearing ↔Scanning ↔Sap ↓Flat back approach ↓Immobility Acute treatment—Fluoxetine: ↑Enclosed arms entries ↔Peeping out ↔Grooming ↔Rearing ↔Scanning ↔Flat-back approach ↔Stretched-attend posture ↔Immobility ↓Open arms entries ↓Time spent in open arms ↓Time spent in center platform ↓Head dipping ↓End-arm activity Chronic treatment—Fluoxetine: ↔Open arm entries ↔Time spent in center platform ↔Time spent in open arms ↔Peeping out ↔Grooming ↔Rearing ↔Scanning ↔Flat-back approach ↔Head dipping ↔Stretch-attend posture ↔End-arm activity ↔Immobility
Bodnoff et al. (1989) [65]	Male Long-Evans rats	Novelty-suppressed feeding Acute/chronic (21 days) treatment Gepirone (4 mg/kg i.p.); Diazepam (2 mg/kg i.p.); Desipramine (10 mg/kg i.p.); Amitriptyline (10 mg/kg i.p.); Mianserin (10 mg/kg i.p.); Fluoxetine (10 mg/kg i.p.); Nomifensine (10 mg/kg i.p.); Buspirone (4 mg/kg i.p.)	Gepirone/Buspirone/Mianserin: ↔ Latency to began eating (acute) ↓ Latency to began eating (chronic) Diazepam: ↓ Latency to began eating (acute and chronic) Desipramine/Amitriptyline/Fluoxetine: ↑ Latency to began eating (acute) ↓ Latency to began eating (chronic) Nomifensine: ↑ Latency to began eating (acute) ↔ Latency to began eating (chronic)
De Vry et al. (1993) [66]	Male/Female Wistar rats	Shock-induced ultrasonic vocalization N = 9–10 per group Gepirone: ‐ 0.3–10 mg/kg i.p. ‐ 1–30 mg/kg p.o.	Dose dependent complete reduction in ultrasonic vocalization in i.p. administration. Dose dependent almost complete (95%) reduction of ultrasonic vocalization in p.o. administration.
Przegaliński et al. (1990) [67]	Male Wistar rats	Forced swimming test + open field test N = 8–10 per group Acute/3 injection in 24 h Gepirone (i.p.): ‐ 2.5 mg/kg; ‐ 5 mg/kg; ‐ 10 mg/kg and ‐ 20 mg/kg i.p.) Ipsapirone, buspirone, 1-PP (i.p.): ‐ 5 mg/kg; ‐ 10 mg/kg; ‐ 20 mg/kg. Proadifen (i.p.): ‐ 50 mg/kg.	Gepirone alone: ↓Immobility time (acute and repeated treatment). ↓Ambulation (20 mg/kg) ↓Rearing Gepirone (5 and 10 mg/kg) + Proadifen: ↓Immobility time (more potent) Buspirone, Ipsapirone, 1-PP: ↔ Immobility time (acute and repeated treatment) Exception: ipsapirone 5 mg/kg—↓immobility time (reapted treatment Gepirone/Buspirone/Ipsapirone (20 mg/kg + Proadifen + 1-PP (4 mg/kg) ↔Immobility time
Chojnacka-Wójcik et al. (1991) [68]	Male Wistar rats	Forced swimming test N = 8–10 per group Gepirone (i.p.) ‐ 2.5 mg/kg; ‐ 5 mg/kg; ‐ 10 mg/kg; ‐ 20 mg/kg. NAN-190 (i.p.): ‐ 0.25 mg/kg; ‐ 0.5 mg/kg. Pindolol (i.p.): ‐ 2 mg/kg; ‐ 4 mg/kg. Spiperone (i.p.): ‐ 0.01 mg/kg; ‐ 0.03 mg/kg. Haloperidol (i.p.): ‐ 0.125 mg/kg; ‐ 0.25 mg/kg. Metergoline (i.p.): ‐ 2 mg/kg; ‐ 4 mg/kg. Ketanserin (i.p.): ‐ 1 mg/kg; ‐ 2 mg/kg. Prazosin (i.p.): ‐ 0.5 mg/kg; ‐ 1 mg/kg. Betaxolol (i.p.): ‐ 4 mg/kg; ‐ 8 mg/kg. ICI 118,551 (i.p.) ‐ 4 mg/kg; ‐ 8 mg/kg. PCA (i.p.): ‐ 20 mg/kg; PCPA (i.p.): ‐ 900 mg/kg.	Gepirone alone: ↓Immobility time (dose-dependent) Gepirone + Metergoline: ↓Immobility time Gepione + Ketanserin: ↓Immobility time Gepirone + Prazosin: ↓Immobility time Gepirone + Betaxolol: ↓Immobility time Gepirone + ICI 119,551: ↓Immobility time Gepirone + PCA: ↓Immobility time Gepirone + PCPA: ↓Immobility time Gepirone + NAN-190 ↔Immobility time Gepirone + Pindolol: ↔Immobility time Gepirone + Spiperone: ↔Immobility time Gepirone + Haloperidol: ↔Immobility time
Benvenga and Leander (1993) [69]	Male Sprague-Dawley rats	Forced swim test N = 6 per group Repeated treatment (24, 3 and 1 h before test) Gepirone (i.p.): ‐ 5 mg/kg; ‐ 10 mg/kg; ‐ 20 mg/kg. Impiramine (i.p.): ‐ 5 mg/kg; ‐ 10 mg/kg; ‐ 20 mg/kg; ‐ 40 mg/kg. 8-OH-DPAT (i.p.): ‐ 0.16 mg/kg; ‐ 0.32 mg/kg; ‐ 0.64 mg/kg; ‐ 1.25 mg/kg. LY228729 (s.c.): ‐ 0.3 mg/kg; ‐ 1 mg/kg; ‐ 3 mg/kg.	Gepirone: ↔Immobility time (5–10 mg/kg); ↓Immobility time (20 mg/kg). Impiramine: ↔Immobility time (5–20 mg/kg); ↓Immobility time (40 mg/kg); ↑Sedation (20–40 mg/kg); 8-OH-DPAT: ↑Immobility time (0.32 mg/kg); ↔Immobility time (0.16 and 0.64 mg/kg); ↓Immobility time (1.25 mg/kg). LY228729: ↔Immobility time (0.3 mg/kg); ↓Immobility time (1–3 mg/kg); ↑Frequency of flat body posture (1–3 mg/kg; short duration, only on first day of testing); ↔Locomotor activity.
Giral et al. (1988) [70]	Male Wistar AF rats	Inescapable footshock N = 8–12 per group Gepirone (i.p.; 5 days treatment): ‐ 0.06 mg/kg; ‐ 0.125 mg/kg. Buspirone (i.p.; 5 days treatment): ‐ 0.5 mg/kg; ‐ 1 mg/kg. 8-OH-DPAT ((i.p.; 5 days treatment): ‐ 0.03 mg/kg; ‐ 0.06 mg/kg; ‐ 0.125 mg/kg; ‐ 0.25 mg/kg. TVXQ 7821 (i.p.; 5 days treatment): ‐ 0.03 mg/kg; ‐ 0.06 mg/kg.	Gepirone: ↓Escape failures 8-OH-DPAT: ↓Escape failures Buspirone: ↓Escape failures TVXQ 7821: ↓;Escape failures
Drugan et al. (1987) [71]	Male Sprague-Dawley rats	Inescapable shock in two-way shuttle-box Gepirone/buspirone (i.p.): ‐ 2 mg/kg ‐ 5 mg/kg Chlordiazepoxide (i.p.): ‐ 5 mg/kg	Gepirone: ↔Latency to escape response Buspirone: ↔Latency to escape response (2 mg/kg) ↓Latency to escape response (5 mg/kg) Chlordiazepoxide: ↓Latency to escape response
Camargo et al. (2008) [72]	Male Wistar rats	Inescapable footshock N = 8 per group (4 groups: well-nourished exposed/non-exposed to shock and malnourished exposed/nonexposed to shock) Gepirone/Chlorodiazepoxide (i.p.): ‐ 2.5 mg/kg ‐ 5 mg/kg; ‐ 7.5 mg/kg.	Gepirone: ↑Escape latencies (animals exposed on inescapable shock in comparison to non-exposed animals) ↑Escape latencies in well-nourished non-exposed rats (5 mg/kg) Chlodiazepoxide: ↑Escape latencies (animals exposed on inescapable shock in comparison to non-exposed animals) ↓Escape latency (5 and 7.5 mg/kg in comparison to saline) ↑Escape latency (malnourished animals in comparison to well-nourished animals)
Barrett et al. (1988) [73]	Male White Carneaux pigeons	Punished response test N = 6 per group Gepirone (i.m): ‐ 0.03 mg/kg; ‐ 0.1 mg/kg; ‐ 0.3 mg/kg; ‐ 1 mg/kg; ‐ 3 mg/kg; ‐ 10 mg/kg.	↑Responces in FI schedule (doses 0.03–3 mg/kg) ↑Responces in FR schedule (doeses 0.3 and 1 mg/kg)

Abbreviations: i.g.—intragastrically; i.m.—intramuscularly; i.v.—intravenous; p.o.—per orally; i.p.—intraperitoneally; s.c.—subcutaneously; (↑)—increase; (↓)—decrease; (↔)—no changes.

## 4. Clinical Development of Gepirone

The earliest clinical investigations assessed the therapeutic potential of the gepirone IR. In an open-label study of patients with MDD, the dose was titrated up to 60 mg/day over six weeks. Improvements were observed in selected items of the Hamilton Depression Rating Scale (HAM-D), particularly items 1, 7, and 8, suggesting an antidepressant effect. Subgroup analyses of patients with melancholic features revealed similar outcomes. In another open-label trial involving patients with atypical depression, divided doses of gepirone up to 90 mg/day significantly reduced mean HAM-D scores compared with baseline. Taken together, these findings provided preliminary evidence for the clinical efficacy of gepirone [74]. Subsequently, Rausch et al. [75] conducted a single-blind study in patients with MDD, in which gepirone was titrated up to 70 mg/day. Statistically significant reductions were observed in the 24-item HAM-D, further supporting the antidepressant potential of gepirone [75].

Beyond depression, gepirone IR was also evaluated in anxiety disorders. In an open-label, six-week study of patients with chronic anxiety, treatment with gepirone (up to 60 mg/day) significantly reduced HAM-A scores (from approximately 25 at baseline to 10 at week 6). These results were confirmed in a double-blind, placebo-controlled trial, where gepirone (15–60 mg/day) produced a greater reduction in HAM-A scores compared to placebo [68]. Harto et al. [76] further assessed gepirone in patients with generalized anxiety disorder (DSM-III criteria). Gradual titration up to 60 mg/day over 42 days resulted in significant improvements on the HAM-A, Clinical Global Impressions-Severity (CGI-S), and Clinical Global Impressions-Improvement (CGI-I) scales [76].

Despite these encouraging early observations of antidepressant and anxiolytic activity [74,75], larger controlled trials produced inconsistent results. Phase II/III studies yielded mixed findings: while some demonstrated significant advantages over placebo, others failed to meet primary efficacy endpoints. Antidepressant effects often emerged only after six weeks of treatment, later than with tricyclic antidepressants (TCA) such as imipramine, and the overall response was weaker and less predictable. In several trials, gepirone IR produced improvements on selected secondary scales (e.g., HAM-D), but not on primary endpoints required for regulatory approval [33]. In addition to these clinical limitations, gepirone IR displayed unfavorable pharmacokinetic properties (see Table 1). Its short half-life led to rapid fluctuations in plasma concentrations, resulting in inconsistent clinical effects. Patients frequently experienced transient benefits after each dose, followed by rapid symptom recurrence. This pharmacokinetic profile necessitated three to four daily doses and was associated with increased adverse events such as dizziness, nausea, and withdrawal-like symptoms between doses.

To address these limitations, an gepirone ER was developed. One of the earliest randomized controlled trials with the extended-release formulation of gepirone enrolled patients with MDD diagnosed according to DSM-III-R criteria who scored ≥20 on the HAM-D17 [77,78]. In this double-blind study, participants were randomized to low-dose gepirone ER (10–50 mg/day), high-dose gepirone ER (20–100 mg/day), or placebo. The dose was tailored individually to each patient based on efficacy and side effects or the absence of both factors. The most robust improvements were observed in the high-dose group, where statistically significant reductions in depressive symptoms were recorded compared with placebo. In contrast, patients receiving low-dose gepirone showed significant improvement only on the Bech-6 subscale at week 6 [77]. A trial with a similar design compared gepirone ER (10–60 mg/day) with imipramine (50–300 mg/day) and the placebo. While imipramine demonstrated efficacy as early on as week 2, gepirone ER achieved comparable outcomes by week 6 but was associated with superior tolerability, as evidenced by fewer discontinuations due to adverse effects (2 vs. 12 for imipramine). The authors noted that the average daily dose of gepirone ER (40 mg) may have been suboptimal, potentially accounting for the slower onset of effect [78]. These findings reinforced prior observations that higher doses of gepirone ER are required to achieve clinically meaningful efficacy [77].

Following initial FDA rejection, additional randomized, placebo-controlled trials were conducted to further evaluate gepirone ER in MDD [79,80,81]. Alpert et al. included patients with comorbid anxiety, while Keller et al. incorporated an open-label lead-in phase (20–80 mg/day) to achieve remission, followed by randomization into a double-blind relapse-prevention phase [79,80]. Bielski et al. [81] described Protocol FKGBE007, a multicenter trial conducted at nine U.S. sites between 2003 and 2004, in which patients initiated treatment at 20 mg/day with titration to 40–80 mg/day by day 14, depending on response and tolerability [81]. Across these studies, gepirone ER demonstrated statistically significant improvements from baseline on the HAM-D17, HAM-D25, MADRS, and CGI scales (*p* ≤ 0.05) [79,80,81]. Moreover, the continuation phase of Keller’s trial confirmed the safety and efficacy of long-term therapy, with gepirone ER reducing relapse rates for up to one year [80]. Subgroup analyses provided additional insights. In Alpert at el. [79], the antidepressant efficacy of gepirone ER was compared in patients with anxious versus non-anxious depression. While overall response rates did not differ significantly between groups, gepirone ER conferred a higher likelihood of remission among patients with elevated baseline anxiety, suggesting potential benefit in anxious depression. However, these results were insufficient to conclusively establish anxiety severity as a moderator of treatment response [80].

Phase II/III development further included large-scale, double-blind, placebo-controlled studies such as CN105-083 and CN105-078, which compared low-dose (10–50 mg/day) and high-dose (20–100 mg/day; individually tailored to the patient) gepirone ER with placebo, and Protocols 134002 and FK-GBE-008, which employed dosing ranges of 20–80 mg/day and 40–80 mg/day, respectively. Although not all trials met every predefined endpoint, gepirone ER demonstrated superiority over placebo on at least one primary efficacy measure in multiple studies, which was considered supportive of efficacy by regulatory authorities [82]. The relapse-prevention study 28709 also contributed to the clinical evidence base: although initial analyses suggested a significant reduction in relapse risk with gepirone ER (23% vs. 34.7%, *p* = 0.024), subsequent FDA reanalysis corrected for patient misclassification and diminished the statistical significance (27% vs. 34.7%, *p* = 0.10) [82].

Despite these advances, not all studies produced positive results. Fabre et al. examined gepirone ER in protocols 134004, 134006, and 134017, evaluating its impact on sexual dysfunction in patients with depression [44,83]. Trials in both atypical depression (134004, 134006) and MDD (134017) found no significant antidepressant effect of gepirone ER compared with placebo or SSRIs when measured by HAM-D25 or MADRS. Nevertheless, gepirone ER produced clinically meaningful improvements in hypoactive sexual desire disorder (HSDD), where SSRIs were ineffective. In women, gepirone ER demonstrated significant benefits by week 8 across all studies [83], and in men, it improved CSFQ scores among nonresponders to antidepressant therapy, independent of mood effects [44] (Table 3). Gepirone ER ultimately received FDA approval for the treatment of MDD in 2023.

## 5. Dosage, Safety, Drug Interactions and Adverse Effects of Gepirone ER

### 5.1. Dosage and Administration

Gepirone ER is available in tablet strengths of 18.2 mg, 36.3 mg, 54.5 mg, and 72.6 mg. Therapy should generally begin with 18.2 mg once daily, taken orally with food at roughly the same time each day. If tolerated, the dose can be gradually increased: to 36.3 mg/day after 4 days, 54.5 mg/day after 7 days, and up to a maximum of 72.6 mg/day after 14 days [20,31].

In patients with mild hepatic impairment (Child-Pugh A), no adjustment is required. For moderate impairment (Child-Pugh B), the daily dose should not exceed 36.3 mg, while severe hepatic impairment (Child-Pugh C) is a contraindication to treatment [31].

Renal dysfunction has a limited effect on gepirone elimination: the drug’s half-life remains stable, but patients with severe renal impairment show roughly a twofold rise in Cmax and prolonged half-life of its metabolite 1-PP [44]. For those with CrCl ≥ 50 mL/min, no adjustment is necessary, while in patients with CrCl < 50 mL/min, the maximum dose should not exceed 36.3 mg/day.

In older adults (≥65 years), systemic exposure (AUC and Cmax) is higher than in younger patients, thus lower maximum daily doses are recommended [31].

### 5.2. Safety Considerations

Gepirone has shown a wide safety margin, with no fatal overdoses reported. Most adverse reactions are mild to moderate and transient, commonly including dizziness, nausea, headache, fatigue, and insomnia [31]. These tend to become more frequent during long-term therapy, potentially burdening patients with MDD.

One notable safety issue is the risk of QT interval prolongation. In studies, gepirone IR at 100 mg/day led to QTc increases of 16–18 msec versus placebo [20]. For this reason, ECG monitoring is advised before and during treatment. Escalation beyond QTc ≥ 450 msec should be avoided due to the risk of arrhythmia [31]. Patients with pre-existing QT prolongation, long QT syndrome, or those receiving QT-prolonging drugs require particular caution. Correction of electrolyte abnormalities (e.g., hypokalemia, hypomagnesemia) is recommended prior to initiation, especially if patients are on diuretics or glucocorticoids [31].

### 5.3. Drug–Drug Interactions

Gepirone is metabolized primarily by CYP3A4, making it highly susceptible to interactions. Strong CYP3A4 inhibitors (e.g., ketoconazole, itraconazole, ritonavir, lopinavir) can raise gepirones’ exposure by ~5-fold, leading to toxic plasma levels. Therefore, co-administration with inhibitors of main cytochrome responsible of biotransformation of this drug is contraindicated. Co-treatment with moderate inhibitors (e.g., erythromycin, diltiazem, fluconazole) and geprione can increase concentration of the second agent approximately 2-fold, thus, it is required to reduce the dosage by 50% [31]. In contrast, CYP3A4 inducers, like rifampin and carbamazepine, markedly reduce exposure. Rifampin was shown to lower Cmax by >90% and AUC by ~95% also decreasing metabolite levels [22]. Therefore, concomitant use therefore should be avoided.

MAOIs, e.g., linezolid, IV methylene blue, are contraindicated due to the risk of serotonin syndrome. It is not allowed to start treatment immediately after each other. At least 14 days should elapse between discontinuation of one drug and initiation of the other. Co-administration with other serotonergic agents (SSRIs, SNRIs, triptans) also increases this risk, requiring monitoring [31].

### 5.4. Adverse Effects

Gepirone ER was generally well tolerated. The most commonly reported adverse effects included headache, nausea, dizziness, and insomnia, while sedation was infrequent [43,44,77,78,79,80,81,82,83]. The drug was not associated with sexual dysfunction, and some subgroups even experienced improvements in sexual function [44,77,79,83]. No clinically significant changes were observed in body weight, laboratory results, physical examination findings, or vital signs [43,79,80,81].

## 6. Clinical Superiority of Gepirone over Other Azapirones

Within the azapirone class, gepirone has emerged as the compound with the greatest clinical utility and evidence base. Although buspirone, tandospirone, and perospirone share the pharmacological hallmark of 5-HT1A receptor partial agonism, their clinical relevance is limited by short half-lives, non-selective receptor interactions, and narrower therapeutic indications [for review see Table 4].

Buspirone was the first azapirone introduced into clinical practice, primarily for generalized anxiety disorder (GAD). While effective in anxiety, its short elimination half-life (~3 h) requires multiple daily dosing, and its modest receptor selectivity—particularly D2 receptor binding—contributes to adverse effects such as dizziness, akathisia, and limited antidepressant efficacy [84,85,86].

Tandospirone, used predominantly in Japan and China for anxiety and depression, exhibits a pharmacological profile similar to buspirone, with partial 5-HT1A agonism and a short half-life (2–3 h). Although it provides anxiolytic benefit, its limited global use and pharmacokinetic constraints reduce its broader clinical impact [27,87].

Perospirone, unlike buspirone and tandospirone, is classified as an atypical antipsychotic. Its receptor profile includes not only partial 5-HT1A agonism but also potent antagonism at D2 and 5-HT2A receptors, which confers efficacy in schizophrenia. However, this broader receptor activity increases the risk of adverse effects associated with antipsychotics, such as sedation, weight gain, and extrapyramidal symptoms [88,89,90].

By contrast, gepirone demonstrates exceptional selectivity for 5-HT1A receptors, functioning as a full agonist at presynaptic autoreceptors and a partial agonist at postsynaptic receptors. This dual mechanism supports both antidepressant and anxiolytic efficacy while minimizing dopaminergic adverse effects. The development of an extended-release (ER) formulation further enhances clinical value by enabling once-daily dosing, stable plasma concentrations, and improved tolerability. Most importantly, gepirone is the only azapirone with FDA approval for the treatment of MDD (as EXXUA^®^), supported by multiple randomized controlled trials demonstrating efficacy, relapse prevention, and favorable tolerability [15,17,18,33].

Taken together, gepirone surpasses buspirone, tandospirone, and perospirone in selectivity, pharmacokinetics, and breadth of validated clinical indications, establishing it as the most clinically relevant and therapeutically advantageous azapirone.

**Table 4:** Pharmacological comparison of gepirone and other azapirones (based on [33,91,92,93,94]).

Feature	Buspirone	Tandospirone	Perospirone	Gepirone ER
**Drug class**	Anxiolytic	Anxiolytic/antidepressant	Atypical antipsychotic	Antidepressant/anxiolytic
**Primary indication**	GAD	Anxiety, depression (Japan/China)	Schizophrenia; schizoaffective disorder	MDD (FDA-approved); anxiety
**Receptor profile**	5-HT1A partial agonist; weak D2 antagonist	5-HT1A partial agonist; minimal D2 activity	5-HT1A partial agonist; D2 and 5-HT2A antagonist	Selective 5-HT1A agonist (full at presynaptic autoreceptors, partial at postsynaptic receptors)
**Half-life**	~3 h	2–3 h	~1.9 h	~6 h
**Cmax**	1.01 ± 0.87 μg/L	3 μg/L (30 mg dosage)	8.8 ng/mL	3.6–4.3 ng/mL
**Tmax**	0.8 h	0.5–2 h	0.8 h	4.8–5.6 h
**AUC**	2.89 ± 3.40 h × μg/L	~115 h × ng/mL	22.0 h × ng/mL	51.8–55.3 h × mg/mL
**Metabolism**	Mainly CYP3A4	Mainly CYP3A4	CYP3A4	CYP3A4, CYP2D6; active metabolites (3′-OH-gepirone, 1-PP)
**Pharmacokinetic limitations**	Short half-life; multiple daily dosing	Short half-life; regional availability only	Very short half-life; antipsychotic-like PK (short half-life, need for frequent dosing, variable plasma levels)	stable plasma levels; avoids peaks/troughs
**Major adverse effects**	Dizziness, headache, limited antidepressant efficacy, mild dopaminergic side effects	Drowsiness, dizziness, rare extrapyramidal symptoms	Sedation, weight gain, extrapyramidal symptoms	Fewer dopaminergic side effects; lower rates of nausea and sexual dysfunction than SSRIs
**Evidence base**	Strong in GAD, weak in depression	Moderate efficacy in anxiety/depression; limited to Asia	Effective in schizophrenia; limited anxiolysis	Robust RCT evidence in MDD; relapse prevention; FDA approval (2023)

## 7. Gepirone ER in Clinical Decision-Making: Practical Algorithm

The introduction of gepirone ER into the therapeutic landscape of MDD offers clinicians a novel alternative, particularly in cases where traditional antidepressants demonstrate limited efficacy or tolerability. A practical, stepwise approach may help to define its optimal placement within current treatment strategies.

First-line therapy for MDD typically involves SSRIs or SNRIs. Following an adequate trial (6–8 weeks at therapeutic doses), patient outcomes can be categorized as full response, partial response, or non-response. Patients achieving remission should continue on maintenance therapy, whereas those with only partial benefit may require dose escalation or augmentation strategies.

Gepirone ER should be considered in patients who fail to achieve satisfactory improvement or who discontinue therapy due to poor tolerability. Several clinical scenarios support its use:

- Inadequate response to SSRIs/SNRIs despite appropriate dose and duration.
- Intolerable side effects of first-line antidepressants, particularly sexual dysfunction, sedation, or sleep disturbances.
- Prominent anxiety symptoms accompanying MDD, where gepirone’s 5-HT_1A_ partial agonism may provide added benefit.
- Patients at risk of benzodiazepine misuse, where gepirone represents a non-addictive anxiolytic alternative.
- Preference for simplified dosing, as the ER formulation allows once-daily administration.

Nonetheless, clinicians should remain cautious in specific populations. Gepirone ER may not be suitable for patients with recurrent migraines (due to headache as a common adverse event), those with prolonged QTc or pre-existing arrhythmia risk, and individuals receiving strong CYP3A4 inhibitors or inducers.

In summary, gepirone ER should be positioned as a second-line or alternative option for patients with MDD who are non-responsive or intolerant to conventional SSRIs/SNRIs, particularly in the presence of comorbid anxiety or SSRI-induced sexual dysfunction. Incorporating gepirone into a structured treatment algorithm may enhance personalization of therapy and expand therapeutic possibilities beyond traditional serotonergic agents.

## 8. Conclusions

Gepirone ER is a valuable addition to the treatment of MDD, addressing limitations of conventional SSRIs and SNRIs such as modest efficacy, delayed onset, and frequent adverse effects including sexual dysfunction and sedation. As a selective 5-HT_1A_ partial agonist, gepirone offers both antidepressant and anxiolytic properties with a more favorable tolerability profile.

Clinically, gepirone ER should be considered a second-line or alternative option for patients with an inadequate response or intolerance to first-line antidepressants, particularly in those with comorbid anxiety, SSRI-induced sexual dysfunction, or risks of benzodiazepine misuse. Its once-daily dosing supports better adherence, though caution is warranted in individuals with headache susceptibility, QT prolongation, or significant CYP3A4 interactions.

By filling key therapeutic gaps, gepirone ER expands opportunities for personalized treatment in MDD. Future studies comparing gepirone with other emerging agents, exploring long-term safety and identifying predictors of response will further clarify its role in modern depression management.

## Figures and Tables

**Table 1:** Pharmacological and Clinical Differences Between Gepirone IR and ER (based on [17,33]).

	Gepirone IR	Gepirone ER
*Dose*	10 mg	20/25 mg daily
*Cmax (ng/mL)*	12.2 ± 6.3	3.6 ± 1.6–4.3 ± 2.8
*Tmax (h)*	1.3 ± 0.9	4.8 ± 1.9–5.6 ± 2.5
*AUC (30) (h × mg/mL)*	54.9 ± 25.6	51.8 ± 27.3–55.3 ± 28.2
*Plasma concentration profile*	High peak-to-trough plasma concentration fluctuation	Steady state plasma concentration
*Dosing interval*	12 h	24 h

**Table 3:** Clinical Trials of Gepirone: Efficacy and Safety in Patients with Depression.

Study	Patient Population	Study Design	Dose(s) of Gepirone	Efficacy Outcomes	Safety Profile
Wilcox et al. 1996 [77]	N = 145 patients with MDD	Randomized, double-blind, placebo-controlled	Low-dose: 10–50 mg/day For 6 weeks. High-dose: 20–100 mg/day For 6 weeks.	Low-dose: No improvement in scales exception: Bech-6 ↓ (*p* < 0.05) week 6 High-dose: Improvements in scales: HAM-D17 ↓ (*p* < 0.05) week 1–6 HAM-D28 ↓ (*p* < 0.01) week 1,4,6 CGI ↓ (*p* < 0.05) week 1,6 Bech 6 ↓ (*p* < 0.05) week 3,4,6	Adverse events were mild to moderate in severity and initially occurred after increasing the dose and subsided over time.
Feiger, 1996 [78]	N = 123 patients with MDD	Randomized, double-blind, placebo-controlled	Gepirone ER: 10–60 mg/day Imipramine: 50–300 mg/day For 8 weeks	Improvements in scales: HAM-D17 ↓ (*p* < 0.05) week 6,8 HAM-D28 ↓ (*p* < 0.05) week 6,8 CGI ↓ (*p* < 0.05) week 8 MADRS ↓ (*p* < 0.01) week 8 Bech ↓ (*p* < 0.05) Week 6,8	Mild and transient side effects.
Feiger et al. 2003 [43]	N = 204 patients with moderate-to-severe MDD	Randomized, double-blind, placebo-controlled	20 mg at day 1 40 mg at day 4 60 mg at day 7 80 mg at day 14 For 8 weeks	Efficacy across primary and secondary parameters: HAM-D17 ↓ (*p* = 0.018) HAM-D25 ↓ (*p* = 0.007) HAM-D item 1 ↓ (*p* = 0.005) MADRS ↓ (*p* = 0.024) Bech-6 ↓ (*p* = 0.007) CGI-S change ↓ (*p* = 0.016) Clinical response and remission at endpoint: HAM-D17 responders ↓ (*p* = 0.059) HAM-D17 remitters ↓ (*p* = 0.017) HAM-D25 responders ↓ (*p* = 0.014) CGI-S responders non-significant (*p* = 0.251)	No serious adverse events occurred.
Alpert et al. 2004 [79]	N = 133 patients with anxious depression	Randomized, double-blind, placebo-controlled	20 mg at day 1 40 mg at day 4 60 mg at day 7 80 mg at day 14 For 8 weeks	Efficacy across primary and secondary parameters: HAM-D17 total scores ↓ (*p* < 0.05) HAM-D17 factor I scores ↓ (*p* < 0.05) HAM-D17 item 12 ↓ (*p* ≤ 0.01) Clinical response and remission at endpoint: HAM-D17 responders ↓ (*p* = 0.035) HAM-D17 remitters ↓ (*p* = 0.002 High vs. low levels of anxiety: HAM-D17 factor I scores ↓ (*p* < 0.001) HAM-D17 responders non-significant (*p* = 0.41) HAM-D17 remitters ↓ (*p* = 0.02)	Dizziness Nausea Headache
Keller et al. 2005 [80]	OL phase: N = 420 patients with MDD Double-blind continuation phase: N = 250 patients who met the criteria	Randomized, placebo-controlled, open-phase for 8-12 weeks, double-blind, placebo-controlled for additional 40-44 weeks	OL phase: 20 mg at day 1 40 mg at day 4 60 mg at day 6 80 mg at day 15 Double-blind continuation phase: Dosage that led to remission in OL phase.	OL phase: HAM-D17 total score ↓ HAM -D17 responders → 68.4% HAM-D17 remitters → 61.4% CGI improved 68.7% Double-blind continuation phase: HAM-D17 total score ↓ HAMD Item 1 ↓ CGI change score ↓ Clinical response and remission at endpoint: HAM-D17 responders → 71.8% HAM-D17 remitters → 64.5%	OL phase: Well tolerated, minimal Nausea Dizziness Headache Insomnia Vertigo Double-blind continuation phase: similar with placebo; Flu Syndrome Headache
Bielski et al. 2008 [81]	N = 248 patients with MDD	Randomized, double-blind, placebo-controlled, parallel-group	20 mg at day 1 40 mg at day 4 40–60 mg at day 8 40–80 mg at day 15	Efficacy across primary and secondary parameters: HAM-D17 total score ↓ (*p* = 0.032) HAM-D28 ↓ (*p* = 0.032) HAM-D Item I ↓ (at week 4,6) MADRS ↓ (*p* = 0.008) CGI-S change ↓ (*p* = 0.015) Bech-6 ↓ (*p* = 0.016) Clinical response and remission at endpoint: HAM-D17 responders → 46% HAM-D28 responders ↑ MARDS responders → 51% CGI-I responders → 48% HAM-D17 remitters → 34.5%	Dizziness Nausea Headache
CN105-083 [82]	N = 112 patients with moderate-to-severe depression	Randomized, double-blind, placebo-controlled	Low dose:10–50 mg/day High-dose: 20–100 mg/day For 6 weeks and 20 weeks extension	Non-significant (*p* = 0.747)	-
CN105-078 [82]	N = 135 patients with moderate-to-severe depression	Randomized, double-blind, placebo-controlled	Low dose: 10–50 mg/day High dose: 20–100 mg/day For 6 weeks and 20 weeks extension	Non-significant (*p* = 0.362)	-
FKG-GBE-008 [82]	N = 195 patients with moderate-to-severe depression	Randomized, double-blind, placebo-controlled	40–80 mg/day For 8 weeks	Non-significant (*p* = 0.195)	-
134002 [82]	N = 211 patients with moderate-to-severe depression	Randomized, double-blind, placebo-controlled	20–80 mg/day For 8 weeks	Non-significant (*p* = 0.417)	-
28709 [82]	N = 303 in Phase 1; N = 250 in Phase 2; patients with moderate-to-severe depression	Phase 1: Open-label phase 8–12 weeks; Phase 2: Randomized, double-blind, placebo-controlled 40–44 weeks	20–80 mg	Non-significant (*p* = 0.10124)	-
Fabre et al. 2011 [83]	N = 921 female patients in 3 trials with MDD/atypical depressive disorder	Randomized, double-blind	Gepirone ER: 20 mg at day 1 40–60 mg at day 4 60–80 mg at day 14 Fluoxetine (134004, 134017): 20 mg/day start 40 mg/day at week 2 Paroxetine (134006): 20 mg/day start 30 mg/day at week 2 40 mg/day at week 3	134004: HAM-D25 no significant DSM-IV at week 2 (p = 0.061); at week 8/EOT (p = 0.278) 134006 HAM-D25 no significant DSM-IV at week 2 (*p* = 0.010) at week 8/EOT (*p* = 0.022) 134017 MARDS no significant DSM-IV at week 2 (*p* = 0.096); at week 8/EOT (*p* = 0.257) Cumulative DSM-IV at week 2 (*p* = 0.0001) at week 8/EOT (*p* = 0.007)	Dizziness Nausea No decreased libido compared to SSRIs
Fabre et al. 2012 [44]	N = 181 male patients with MDD	Randomized, double-blind	Gepirone ER: 40–80 mg/day Fluoxetine: 20–40 mg/day	HAM-D non-significant MADRS non-significant CSFQ Depressed men (*p* = 0.046) HAM-D17 non-responders (*p* = 0.011) week 4 MADRS non-responders (*p* = 0.002) week 4 HAM-D/MADRS responders non-significant	Dizziness Nausea

Abbreviations: (↑)—increase; (↓)—decrease.

## Data Availability

This review article relies exclusively on previously published data, fully cited in the bibliography. The original datasets used in the cited studies were not accessed or analyzed by the authors.
